# Determining Appropriate Screening Tools and Cutoffs for Cognitive Impairment in the Chinese Elderly

**DOI:** 10.3389/fpsyt.2021.773281

**Published:** 2021-12-02

**Authors:** Shaowei Zhang, Qi Qiu, Shixing Qian, Xiang Lin, Feng Yan, Lin Sun, Shifu Xiao, Jinghua Wang, Yuan Fang, Xia Li

**Affiliations:** Shanghai Mental Health Center, Shanghai Jiao Tong University School of Medicine, Shanghai, China

**Keywords:** Mini-Mental State Exam (MMSE), Montreal Cognitive Assessment (MoCA), mild cognitive impairment (MCI), dementia, China

## Abstract

**Background:** The Mini-Mental State Examination (MMSE) and Montreal Cognitive Assessment (MoCA) are the most commonly used tools for cognitive impairment screening. The present study aimed to investigate the ability of the MOCA and MMSE to differentiate between cognitively normal elderly individuals, MCI patients and dementia patients at different ages and education levels and to establish the optimal cutoff scores of the MoCA and MMSE for MCI and dementia in the Chinese elderly.

**Methods:** A total of 2,954 Chinese elderly individuals, including 1,746 normal controls, 599 MCI patients and 249 dementia patients, were consecutively recruited in the study. The optimal cutoffs for MoCA and MMSE were determined using receiver operating characteristic (ROC) analysis among the different age and education levels in the three groups. Furthermore, comparison of ROC curves were made to evaluate the performances of the two tests.

**Results:** The area under the curve(AUC) of the MoCA (0.82) for detecting MCI was significantly higher than that of the MMSE (0.75) (*P* < 0.001). When the sample was divided according to age and education level, the AUC of the MoCA (0.84) was higher than those of the MMSE (0.71) for MCI (*P* < 0.001) in the younger and more highly-educated groups. The optimal cutoff scores of the MoCA for the groups aged ≤ 75 years old and education ≤ 6 years, aged > 75 years old and education ≤ 6 years, aged ≤ 75 years old and education > 6 years, aged > 75 years old and education > 6 years in screening for MCI were identified as 19.5, 15.5, 24.5 and 24.5, respectively, and the optimal cutoff scores for dementia were 18.5, 10.5, 18.5 and 20.5, respectively. For MMSE in the above four groups, the cutoff scores to detect MCI were 26.5, 22.5, 28.5 and 26.5, respectively, and the optimal cutoff scores for dementia were 23.5, 19.5, 23.5 and 23.5, respectively.

**Conclusion:** Compared to MMSE, the MoCA is more suitable for discriminating MCI in younger and more highly educated elderly Chinese individuals. However, the MMSE has advantage over MoCA in screening MCI in individuals with lower education levels and the older groups of Chinese elderly.

## Introduction

With an aging population, the number of patients with dementia has increased worldwide. By 2050, 115 million people will have dementia (1). Mild cognitive impairment (MCI) is a transitional level between the normal state of the brain and dementia (2), and the prevalence of MCI in adults aged ≥ 65 years is 10–20%. Although MCI is associated with a high risk of dementia, it sometimes remains normal or slightly decreases cognitive function without any notable interference in daily life activities (3). With the progress of technology and the development of a social civilization, several interventions, such as combined cognitive and physical exercise (4), have already been shown to be more effective in preventing greater reductions in cognition early in the course of the disease (5, 6). If the progression of MCI to dementia could be delayed by 5 years, the prevalence of dementia would drop by 43% by 2050 (1). Thus, MCI has become a novel topic in current research.

Although timely diagnosis is important, distinguishing MCI from normal age-related cognitive decline is a challenging task for clinicians (3, 5, 7). Although the National Institute on Aging and Alzheimer's Association (NIA-AA) created an AT(N) diagnosis scheme (8, 9), these biomarkers are expensive and invasive (10) and cannot be used widely for screening. A range of cognitive assessment tasks that are sensitive to cognitive impairment are available, but many are domain specific and time-consuming in actual clinical practice (11). Thus, community screening for MCI is a great challenge globally, and this is no different in China (12).

Considering that screening may lead to patients simply being treated for longer periods, with additional costs to the government but with no benefit to the patient, the United States Preventive Services Task Force and the UK National Screening Committee do not recommend dementia screening (5, 13). The fear, loss and stigma that are associated with dementia also discourage many patients from choosing cognitive assessments (14). In China, we have the same problem. However, in China, the government planned to establish a prevention and treatment service network for dementia. The National Health Commission of the People's Republic of China printed *Exploring the Work Plan of the Special Service for the Prevention and Treatment of Dementia (*http://www.nhc.gov.cn/), which aims to achieve 80% coverage of cognitive function screening in the elderly by 2022.

The Mini-Mental State Exam (MMSE) and Montreal Cognitive Assessment (MoCA) are commonly used cognitive screening scales (15). The MMSE is a 30-question assessment of cognitive function that evaluates attention and orientation, memory, registration, recall, calculation, language and the ability to draw a complex polygon (16). Traditionally, the MMSE has been used to distinguish between patients with cognitive impairment and dementia by using a 23/24 cutoff value (17). Then, the MoCA is a recently developed cognitive screening test that is used to distinguish between normal aging and mild cognitive impairment (MCI) by using a cutoff score of 26 (18). Most studies have reported consistent results that the MoCA provides higher diagnostic accuracy and lower specificity than the MMSE for MCI detection (19). However, some studies have suggested that factors such as sociocultural factors, age and years of education may influence individual scores (20). Therefore, older and/or less-educated individuals are at higher risk of false-positive results when using the current cutoff values (21).

In the context of demographic change, the older population, especially the middle-old and oldest-old (75 years or older) populations, is increasing. As these elderly individuals tend to have poor health status, cognitive function needs early attention. In addition, the education levels of the elderly in China and in some developing countries are generally low. Therefore, it is important to develop local standards for each population and a set of evaluations for elderly (≥65 years of age) and very elderly (75 years or older) individuals (22). Based on a long-term community-based cohort study that was conducted by our team, this study aimed to address this problem, compare the ability of the MOCA and MMSE to distinguish MCI from dementia/normal aging at different educational levels and ages, and determine the corresponding cutoff values.

## Materials and Methods

### Participants

The participants for this study were recruited from both the National Pillar Program which is associated with 15 research centers in 8 cities (23) and 4 different communities in Shanghai, and Major Research Program of the Shanghai Clinical Medical Center for Mental Disorders. This study was approved by the Institution's Ethical Committee of Shanghai Mental Health Center, Shanghai Jiao Tong University School of Medicine, and written informed consent was obtained from all subjects and/or their legal guardians. The participants were placed into the analysis if they had been classified as having normal cognition, MCI, or dementia. The included sample consisted of 2,954 participants, with 599 meeting the Petersen criteria for MCI, 249 diagnosed with dementia, and 1,746 people with normal cognition.

### Assessment Protocol

The participants completed an entire assessment, which included history taking, cognitive assessment, and neuropsychological evaluation. The investigators were uniformly trained and were qualified medical staff from the departments of psychiatry and geriatrics. The Chinese version of the Mini Mental State Examination (MMSE) (24) and Beijing version of the Montreal Cognitive Assessment (MoCA-BJ), which were validated in Chinese population (25), were used as part of the cognitive assessments of all participants. The education levels were defined by the number of years of education attained, and individuals were considered to be illiterate when they reported they could not read and write or had <1 year of education.

### Clinical Diagnoses

The clinical diagnoses were made based on the results of patient histories, systematic neuropsychological tests, physical examinations, head CTs or MRIs, routine blood tests, examinations of hepatorenal function, folic acid and vitamin B12 levels, thyroid function tests, and syphilis antibody tests. A diagnosis of MCI was made according to the Petersen criteria (26), and dementia was diagnosed according to Diagnostic and Statistical Manual of Mental Disorders (4th ed., DSM–IV) criteria (27). In addition, these deficits must include a significant impairment in social or occupational functioning and constitute a change from a previous level of performance.

### Analysis

The statistical analysis were performed using SPSS software (ver. 20.0; SPSS, Inc., Chicago, IL, USA). Analysis of variance (ANOVA) was used to compare the MoCA scores with the samples in different clinical groups. The MMSE scores were also compared using ANOVA. The significance level was set at 1% (*p* < 0.01). SigmaPlot 12.5 was used to identify the differences in the receiver operating characteristic (ROC) curve analyses, which provided scores for the area under the curve, sensitivity and specificity among the different age and education levels in the three groups.

## Results

### Demographic Situation

Among the 2,954 participants in this study, 974 (37.5%) were aged ≥ 75 years old, 860 (33.2%) had 6 years of education or fewer, and 1,121 (43.2%) were male. Based on their clinical diagnoses, the participants were divided into three groups: 599 (23.1%) participants with MCI, aMCI accounted for 82.97% and vMCI accounted for 17.03%. 249 (9.6%) participants with dementia, including Alzheimer's disease (65.86%), Vascular dementia (29.32%) and other dementias (4.82%). 1746 (67.3%) participants who were cognitively normal (Table 1). The normal cognition group was significantly younger and was more educated than the dementia and MCI groups. The dementia group was significantly older than the other groups. The average education levels in the dementia and MCI groups were similar. The gender distributions and the ratio of white-collar workers were significantly different between the normal cognition group and the other two groups but did not differ between the MCI and dementia groups. The ratio of comorbidities such as hypertension, diabetes, hyperlipidemia, hematencephalon, and cerebral infarction did not differ among the three groups (Table 2).

**Table 1:** All types of dementia and MCI.

**Types**	***N***	**Scale (%)**
aMCI	497	82.97
vMCI	102	17.03
All MCI	599	100
AD	164	65.86
VD	73	29.32
Other dementias	12	4.82
All dementias	249	100

*MCI, mild cognitive impairment; aMCI, amnestic mild cognitive impairment; vMCI, vascular mild cognitive impairment; AD, Alzheimer's disease; VD, Vascular dementia*.

**Table 2:** Demographic and clinical data of the participants.

**Variables**	**Normal**	**MCI**	**Dementia**	**F/**χ^2^****	***P***
	**(*n* = 1,746)**	**(*n* = 599)**	**(*n* = 249)**		
Age, year	70.46 ± 6.98	74.03 ± 7.29	75.23 ± 10.12	82.225	<0.001
Education, year	10.07 ± 4.75	7.19 ± 5.45	6.45 ± 5.67	109.205	<0.001
Male,n(%)	809 (46.4%)	218 (36.4%)	94 (37.9%)	21.353	<0.001
MMSE	26.95 ± 3.46	22.25 ± 5.19	13.15 ± 7.61	985.521	<0.001
MoCA	23.79 ± 5.15	17.75 ± 5.56	8.73 ± 5.91	1013.048	<0.001
White-collar workers, *n* (%)	930 (53.3%)	212 (35.4%)	93 (37.3%)	69.607	<0.001
Hypertension, *n* (%)	292 (16.7%)	98 (16.4%)	36 (14.5%)	1.411	0.494
Diabetes, *n* (%)	85 (4.9%)	32 (5.3%)	9 (3.6%)	1.46	0.482
Hyperlipidemia, *n* (%)	26 (1.5%)	12 (2%)	2 (0.8%)	2.242	0.298
Hematencephalon, *n* (%)	37 (2.1%)	6 (1%)	1 (0.4%)	9.236	0.55
Cerebral infarction, *n* (%)	21 (1.2%)	11 (1.8%)	4 (1.6%)	0.837	0.658

*MMSE, Mini-Mental State Examination; MoCA, Mini-Mental State Montreal Cognitive Assessment; MCI, mild cognitive impairment*.

The mean scores of the MMSE among the normal cognition group, MCI and dementia groups were 26.95, 22.25, and 13.15, respectively. The mean scores of the MoCA among the normal cognition group, MCI and dementia groups were 23.79, 17.75, and 8.73, respectively. The scores differed significantly between any two groups.

### Cutoffs and Area Under the Curve (AUC) of the MoCA and MMSE in All Subjects

**Figure 1:**
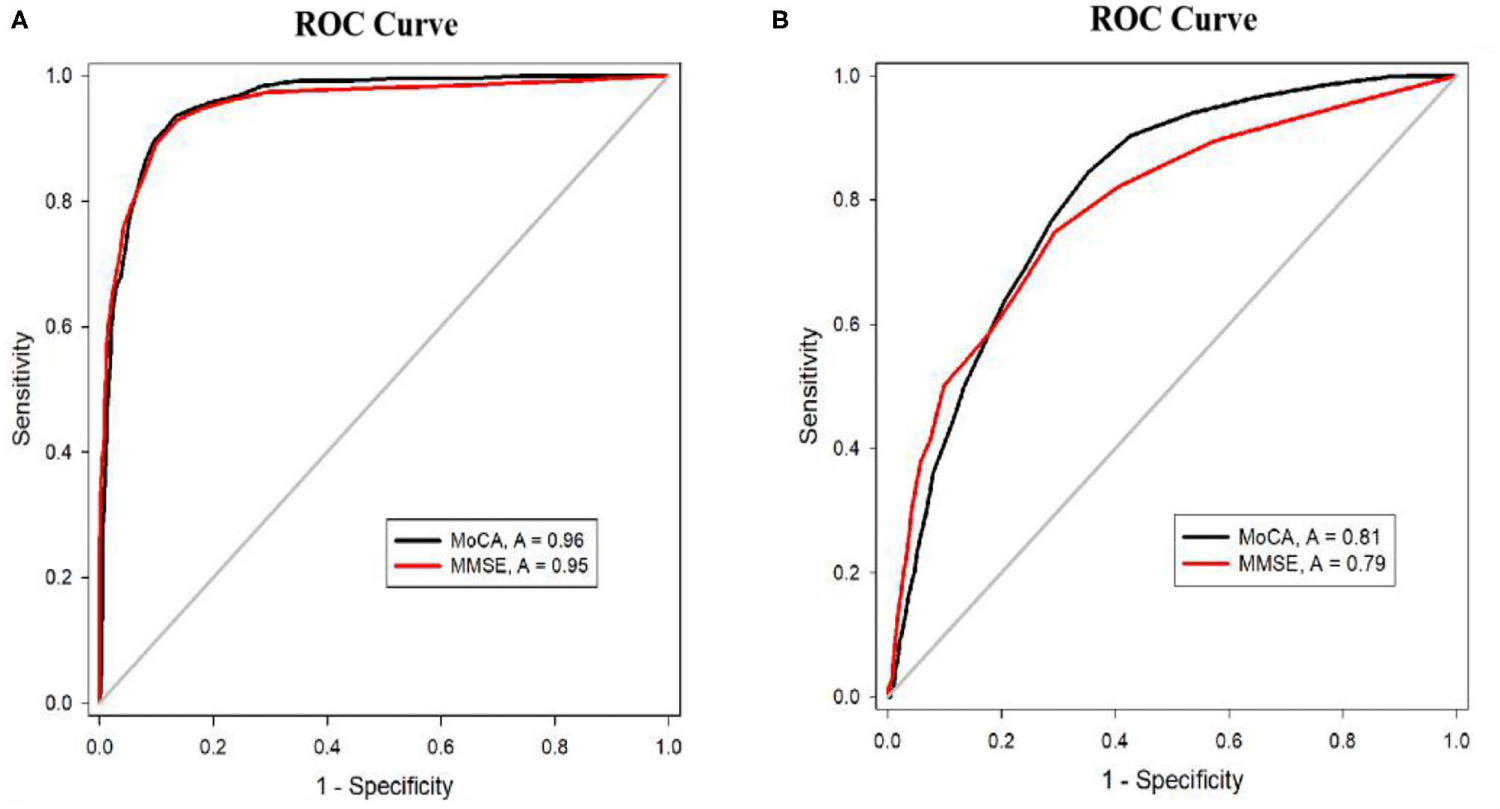
**(A)** ROC curves for MoCA and MMSE for the detection of dementia. **(B)** ROC curves for MoCA and MMSE for the detection of MCI.

The AUCs of both the MoCA and MMSE for detecting dementia were >0.9 (Figure 1A), and showed similar levels of performance. The AUC of the MoCA for detecting MCI was significantly higher than the AUC of the MMSE (*P* < 0.001) (Figure 1B). The cutoffs of the MMSE below 26.5 (sensitivity = 75% and specificity = 71%) and scores below 23.5 on the MoCA (sensitivity = 85% and specificity = 65%) suggested the presence of MCI, while scores below 23.5 (sensitivity = 93% and specificity = 86%) on the MMSE and 18.5 on the MoCA (sensitivity = 96% and specificity = 87%) indicated the presence of dementia.

### Comparison of the Screening and Diagnostic Abilities of the MoCA and MMSE by Age Group

The performance of the MMSE and MoCA were analyzed for the entire sample based on education levels and age bands. The sample was divided into two age groups according to the reported number of years of age.

#### Age Group 1: <75 Years

**Figure 2:**
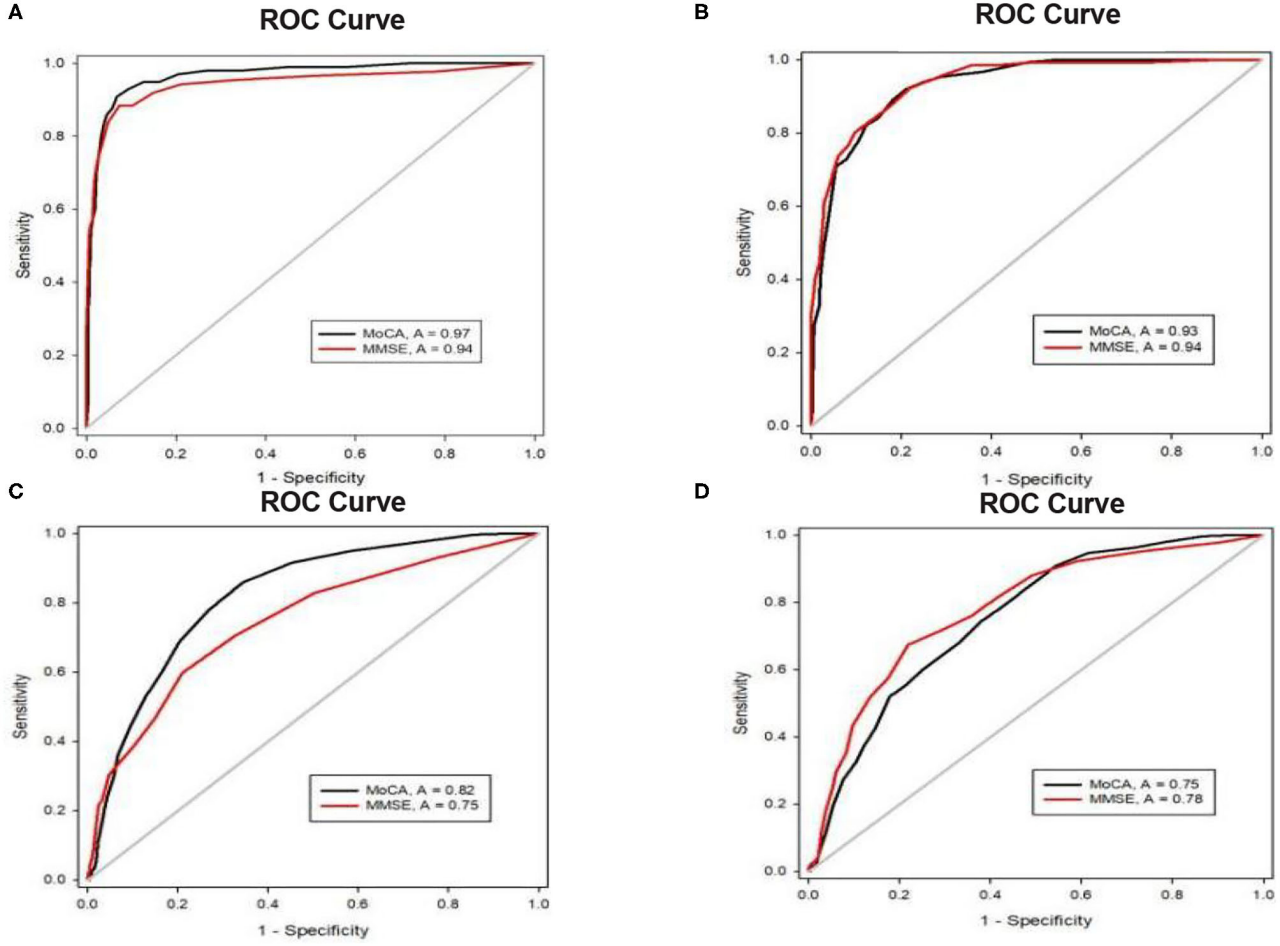
**(A)** ROC curves for the MoCA and MMSE for the detection of dementia in the elders <75 years. **(B)** ROC curves for the MoCA and MMSE for the detection of dementia in the elders ≥ 75 years. **(C)** ROC curves for the MoCA and MMSE for the detection of MCI in the elders <75 years. **(D)** ROC curves for the MoCA and MMSE for the detection of MCI in the elders ≥ 75 years.

In this group, 1,223 participants had normal cognition, and 299 met the criteria for MCI. Dementia was diagnosed in 89 participants. Both the MMSE and MoCA were used to distinguish between normal cognition and dementia, and the AUCs for detecting dementia were all higher than 0.94. The AUC of the MoCA (0.82) for detecting MCI was notably higher than that of the MMSE (0.75), as determined by Sigmaplot (÷2 = 44.32, *p* < 0.001), and the 95% confidence intervals (CI) for the AUCs of the MoCA (0.90–0.95) and MMSE (0.71–0.79) did not overlap (Table 3; Figure 2), which indicates that the ability of the MoCA to detect MCI was significantly better than that of the MMSE.

**Table 3:** MMSE and MoCA data of the normal, MCI and dementia groups by age band.

**Variable**	**Normal**	**MCI**	**Dementia**	**AUC (95%CI)**	
**Age, year**				**Normal vs. MCI**	**Normal vs. Dementia**
<75	*N* = 1,223	*N* = 299	*N* = 98		
MMSE	27.67 ± 2.69	24.43 ± 4.49	14.37 ± 8.28	0.75 (0.71:0.79)	0.94 (0.91:0.98)
MoCA	24.86 ± 4.28	19.65 ± 4.71	9.52 ± 6.43	0.82 (0.80:0.85)^**^	0.97 (0.95:0.98)
≥75	*N* = 523	*N* = 300	*N* = 151		
MMSE	25.23 ± 4.36	20.33 ± 5.00	12.40 ± 7.09	0.78 (0.74:0.82)^**^	0.94 (0.92:0.96)
MoCA	21.29 ± 6.06	15.85 ± 5.71	8.21 ± 5.51	0.75 (0.72:0.79)	0.93 (0.91:0.95)

*MMSE, Mini-Mental State Examination; MoCA, Mini-Mental State Montreal Cognitive Assessment; MCI, mild cognitive impairment; AUC, area under the curve; CI, confidence interval. Bold indicates best performed for that indication; N = people who had complete data for all task*.

** *p < 0.001*.

#### Age Group 2: ≥ 75 Years

Among the 974 participants in this age band, 523 had normal cognition, MCI was detected in 300 people, and dementia was diagnosed in 151 people. Both the MMSE and MOCA could significantly distinguish between NC and dementia. The AUC of the MMSE (0.78) for detecting MCI was higher than that of the MoCA (0.75). Although the 95% CI of the AUC for the MMSE (0.74–0.82) overlapped with that for the MoCA (0.72–0.79), the paired analysis showed that there was a significant difference between the AUC of the MMSE and that of the MoCA (÷2 = 17.88, *p* < 0.001), which indicates that the ability of the MMSE to detect MCI was better than that of the MoCA in this age group.

### Comparison of the Screening and Diagnostic Abilities of the MoCA and the MMSE by Education

#### Education Group 1: ≤ 6 Years of Education

**Figure 3:**
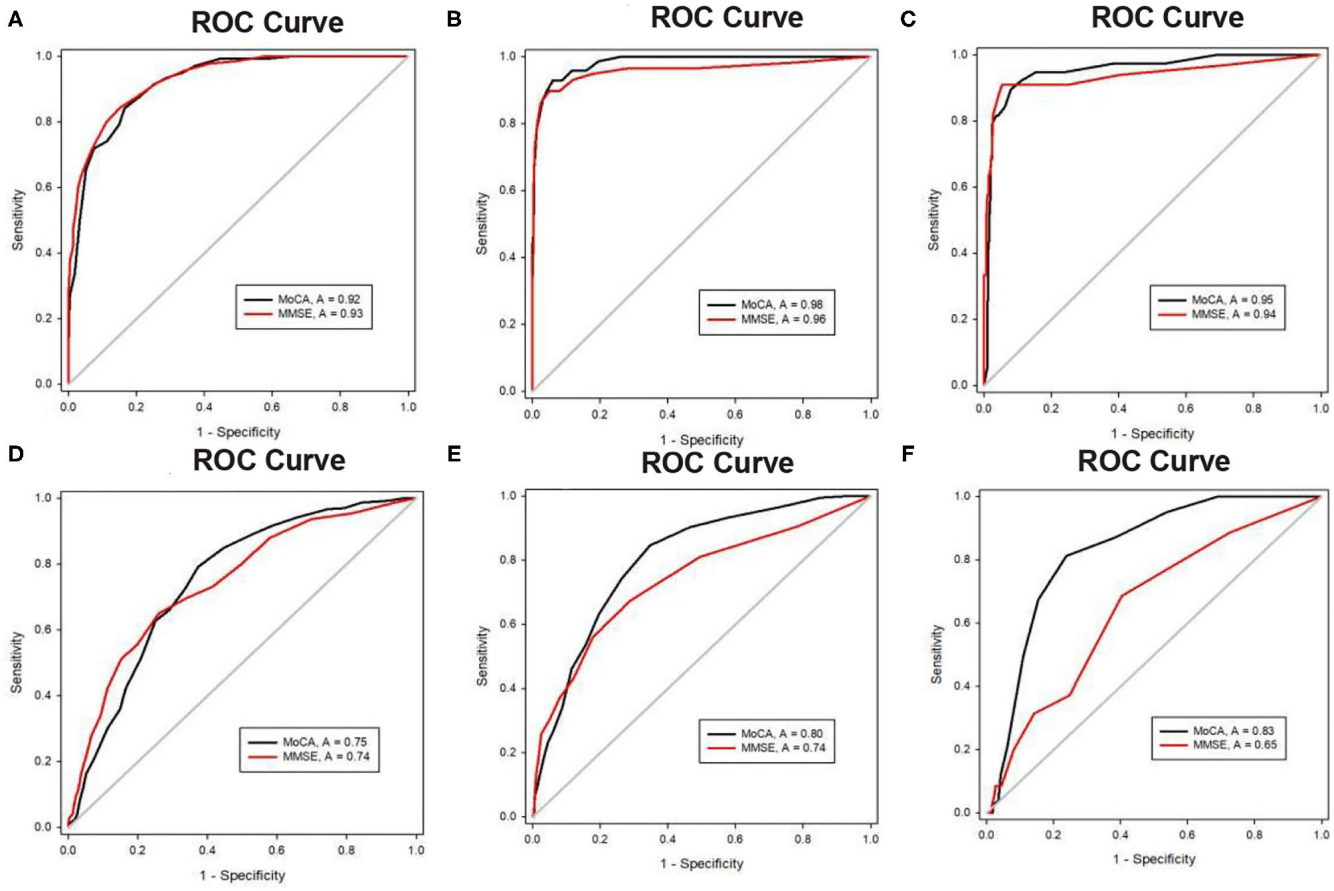
**(A)** ROC curves for MoCA and MMSE for the detection of dementia in elders with ≤ 6 years of education. **(B)** ROC curves for MoCA and MMSE for the detection of dementia in the elders with 7–12 years of education. **(C)** ROC curves for MoCA and MMSE for the detection of dementia in the elders with > 2 years of education. **(D)** ROC curves for MoCA and MMSE for the detection of MCI in the elders with ≤ 6 years of education. **(E)** ROC curves for MoCA and MMSE for the detection of MCI in the elders with 7–12 years of education. **(F)** ROC curves for MoCA and MMSE for the detection of MCI in elders with > 12 years of education.

A total of 423 participants had normal cognition, and 298 met the criteria for MCI in this band. Dementia was diagnosed in 139 participants. The results indicated that the MMSE and MoCA both distinguished between normal cognition and dementia significantly with AUCs > 0.9. There were no significant differences between the MMSE and MoCA for screening MCI or dementia (Table 4; Figure 3).

**Table 4:** MMSE and MoCA data for the normal, MCI and dementia groups by education band.

**Variable**	**Normal**	**MCI**	**Dementia**	**AUC (95%CI)**	
**Education, year**				**Normal vs. MCI**	**Normal vs. Dementia**
≤ 6	*N* = 423	*N* = 298	*N* = 139		
MMSE	24.93 ± 4.21	20.50 ± 5.00	12.73 ± 6.96	0.74 (0.71:0.78)	0.93 (0.91:0.95)
MoCA	19.48 ± 5.83	14.44 ± 4.89	7.97 ± 5.10	0.75 (0.71:0.79)	0.92 (0.90:0.94)
7–12	*N* = 803	*N* = 195	*N* = 70		
MMSE	27.90 ± 2.36	25.13 ± 3.77	13.41 ± 8.25	0.74 (0.69:0.79)	0.96 (0.92:0.99)
MoCA	25.07 ± 3.69	20.56 ± 4.26	9.23 ± 6.27	0.80 (0.77:0.84)^**^	0.98 (0.97:0.99)
>12	*N* = 502	*N* = 101	*N* = 38		
MMSE	28.14 ± 2.55	27.17 ± 2.48	14.61 ± 8.96	0.65 (0.56:0.75)	0.94 (0.87:0.99)
MoCA	25.45 ± 4.38	22.31 ± 2.87	10.61 ± 7.52	0.83 (0.79:0.87)^**^	0.95 (0.92:0.98)

*MMSE, Mini-Mental State Examination; MoCA, Mini-Mental State Montreal Cognitive Assessment; MCI, mild cognitive impairment; AUC, area under the curve; CI,confidence interval. Bold indicates best performed for that indication; N = people who had complete data for all task*.

** *p < 0.001*.

#### Education Group 2: Education of 7–12 Years

A total of 1,068 people had 7–12 years of education, among whom 803 had normal cognition, 195 had MCI, and 70 had dementia. The AUC of the MMSE for detecting dementia was 0.96, and the AUC of the MoCA was 0.98. The AUC of the MoCA (0.80) for detecting MCI was higher than that of the MMSE (0.74). Although the 95% CI of the AUC for the MoCA (0.77–0.84) overlapped with that for the MMSE (0.69–0.79), the paired analysis showed that there was a significant difference between the AUC of the MoCA and that of the MMSE (χ2 = 12.69, *p* < 0.001), which indicated that the ability of the MoCA to detect MCI was better than that of the MMSE in this education group.

#### Education Group 3: >12 Years of Education

Among the 641 people with over 12 years of education, 502 had normal cognition, 101 met the criteria for MCI, and 38 were diagnosed with dementia. Both the MMSE and MoCA could distinguished significantly between normal cognition and dementia with high predictions. However, similar to Education Band 2, there were no significant differences between the AUCs of the MMSE and MoCA. However, the AUC of the MoCA (0.83) for detecting MCI was notably higher than that of the MMSE (0.75) by test (χ2 = 44.32, *p* < 0.001), and the 95% confidence intervals (CI) for the AUCs of the MoCA (0.79–0.87) and MMSE (0.56–0.75) did not overlap, which indicates that the ability of the MoCA to detect MCI was significantly better than that of the MMSE.

### Comparison of the Screening and Diagnostic Abilities of the MoCA and the MMSE by Education Level Within Each Age Group

The sample was divided into education groups based on the reported number of years of education and age.

#### Age ≤ 75 Years and Education ≤ 6 Years Group

In this group, the results in Table 5 indicated that the AUC of the MoCA (0.74) for detecting MCI was notably higher than that of the MMSE (0.67) by test (χ2 = 12.6, *p* < 0.001), which mean that the ability of the MoCA to detect MCI was significantly better than that of the MMSE. The MMSE cutoff of 26.5 distinguished MCI from normal cognition, and the cutoff scores of the MoCA (19.5) best separated normal cognition and MCI and suggested high sensitivity and specificity.

**Table 5:** Cut-off scores, sensitivities, specificities and AUCs (95% CI) of the MoCA and MMSE between normal cognition, MCI and dementia by different educational levels within each age group.

**Variable**		**Normal**	**MCI**	**Dementia**	**Normal vs. MCI**				**Normal vs. Dementia**			
					**Sensitivity**	**Specificity**	**Cut-off**	**AUC (95%CI)**	**Sensitivity**	**Specificity**	**Cut-off**	**AUC (95%CI)**
Age ≤ 75 years and		*N* = 232	*N* = 114	*N* = 26								
education ≤ 6 years	MMSE	25.75 ± 3.58	23.19 ± 4.68	16.33 ± 7.23	75.00	52.17	26.5	0.67 (0.61:0.73)	87.50	78.26	23.5	0.90 (0.85:0.96)
	MoCA	20.98 ± 5.10	16.91 ± 4.68	10.62 ± 5.73	72.48	68.53	19.5	0.74 (0.68:0.79)^**^	92.31	76.29	18.5	0.91 (0.87:0.96)
Age > 75 years and		*N* = 191	*N* = 184	*N* = 113								
education ≤ 6 years	MMSE	23.33 ± 4.53	18.83 ± 4.45	11.95 ± 6.68	81.14	61.90	22.5	0.77 (0.72:0.82)^*^	84.68	83.60	19.5	0.92 (0.89:0.95)
	MoCA	17.66 ± 6.15	12.91 ± 4.36	7.36 ± 4.77	75.54	62.30	15.5	0.73 (0.68:0.78)	77.88	89.53	10.5	0.90 (0.87:0.94)
Age ≤ 75 years and		*N* = 981	*N* = 183	*N* = 70								
education > 6 years	MMSE	28.25 ± 2.01	25.04 ± 3.46	13.72 ± 8.61	74.47	57.49	28.5	0.71 (0.65:0.75)	88.52	97.03	23.5	0.95 (0.90:0.99)
	MoCA	25.79 ± 3.48	20.68 ± 4.16	9.14 ± 6.70	81.42	74.52	24.5	0.84 (0.81:0.87)^**^	90.00	97.35	18.5	0.97 (0.95:0.99)
Age > 75 years and		*N* = 324	*N* = 113	*N* = 38								
education > 6 years	MMSE	27.01 ± 3.30	25.95 ± 3.68	14.10 ± 8.36	64.91	70.23	26.5	0.69 (0.62:0.77)	90.00	88.84	23.5	0.94 (0.89:0.99)
	MoCA	23.49 ± 4.80	20.68 ± 4.16	10.76 ± 6.45	86.37	53.87	24.5	0.72 (0.67:0.77)	84.21	89.47	20.5	0.94 (0.91:0.97)

*MMSE, Mini-Mental State Examination; MoCA, Mini-Mental State Montreal Cognitive Assessment; MCI, mild cognitive impairment; AUC, area under the curve; CI, confidence interval. Bold indicates best performed for that indication; N = people who had complete data for all task*.

* *p < 0.005*,

** *p < 0.001*.

The cutoff scores of the MMSE (23.5) and MoCA (18.5) for dementia all exhibited higher levels of sensitivity and specificity, while the AUC values were > 0.9. However, the AUC values were not significantly different between the MMSE and MoCA for screening dementia.

#### Age > 75 Years and Education ≤ 6 Years Group

The AUC of the MMSE (0.77) for detecting MCI was higher than that of the MoCA (0.73) according to the results (χ2 = 5.72, *p* < 0.05). The results in Table 5 indicate that an MMSE cutoff of 22.5 and MoCA cutoff of 15.5 most accurately separated normal cognition and MCI.

The ROC analyses revealed that the MMSE and MoCA had good accuracies for discriminating normal cognition from dementia with high sensitivities (MMSE = 84.68 and MoCA = 77.88) and specificities (MMSE=83.60 and MoCA = 89.53) in the group when considering low cutoff scores (MMSE=19.5 and MoCA=10.5). Both the MMSE and MoCA had AUCs > 0.9 when differentiating between dementia and normal cognition and had similar abilities to detect dementia.

#### Age ≤ 75 Years and Education > 6 Years Group

The AUC of the MoCA (0.84) for detecting MCI was notably higher than that of the MMSE (0.71) by test (χ2 = 36.04, *p* < 0.001), which means that the ability of the MoCA to detect MCI was significantly better than that of the MMSE in this group. The cutoff scores of the MoCA (24.5) suggested high sensitivity and specificity, which best separated normal cognition and MCI.

The cutoff score of the MMSE (23.5) distinguished dementia from normal cognition, and the cutoff score of the MoCA (18.5) best separated normal cognition and dementia, which suggested high sensitivities and specificities. Both the MMSE and MoCA had AUCs > 0.9 for differentiating between dementia and normal cognition, but the AUC values were not different between the MMSE and MoCA.

#### Age > 75 Years and Education > 6 Years Group

An MMSE cutoff of 26.5 distinguished MCI from normal cognition, and the cutoff scores of MoCA (24.5) best separated normal cognition and MCI, which suggested high sensitivities and specificities. The AUC values were not different between the MMSE and MoCA for separating normal cognition and MCI.

Although there were no differences between the AUCs for the MMSE and MoCA, both were significant for dementia vs. normal cognition (>0.9). The cutoff scores (MMSE = 23.5 and MoCA = 20.5) for dementia had high sensitivities and specificities.

## Discussion

The present study aimed to investigate the ability of the MOCA and MMSE to differentiate between cognitively normal elderly individuals, MCI patients and dementia patients with different ages and education levels. Another aim was to establish the optimal cutoff scores of the MoCA and MMSE for MCI and dementia in the elderly. The results indicated that there were significant differences in age, education and gender between the cognitively normal (CN) and dementia and MCI groups, which were broadly in line with the expectations from previous work that lower levels of education and aging are major risks for neurodegenerative diseases (28, 29). Previous studies have found that the incidence of dementia appears to be similar between males and females, but the prevalence differs (30). Females made up the majority of the MCI and dementia groups at the higher ages, which may be due to the survival differences (31). Our results support that white-collar workers made up the majority in the cognitively normal group, which may be because blue-collar workers, whose jobs are less intellectually demanding, are at a disadvantage compared with white-collar workers in terms of cognitive function. Second, blue-collar work is related to low income, which is associated with poor housing conditions, nutrition, and social environment, potentially giving rise to cognitive impairment (32).

The ROC analyses revealed that the MoCA and MMSE had similar accuracies for identifying dementia, but the MMSE had low accuracy for distinguishing the normal elderly from those with MCI, which is consistent with the results of previous research (33). One possible reason may be that the cognitive domains that are assessed by the MOCA, such as executive function and visuospatial ability, may influence early MCI (34). Because the MMSE is designed to screen for dementia, there are no such domains.

Considering that age and education level are the factors that affect cognitive function, we need to consider their influences on the MOCA and MMSE scores (21, 35). When the sample was divided according to age and education level, a number of trends became evident. Age is considered to be the single greatest risk factor for dementia and other neurodegenerative illnesses. Some researchers have estimated that the prevalence of dementia in the 85 year old and older group was 28.5%, which was more than twice that of the 75- to 84 year old cohort (36). As the age increased, the MMSE and MOCA scores declined not only in the normal cognitive group but also in the MCI and dementia groups, which reflected the negative correlation between age and cognitive decline. The current study suggests that the MOCA reflects MCI more sensitively than the MMSE, and that the MOCA can be used to assess early cognitive decline (37). However, these studies have mainly focused on groups that were younger than 75 years, while there are few relevant articles on older groups (38). In this study, the ability of the MoCA to detect MCI was significantly better than that of the MMSE in groups aged under 75 years. However, for elderly individuals over 75 years, the ability of the MMSE to detect MCI was better than that of the MoCA. The results suggested that the use of the MoCA for MCI screening is more sensitive for younger individuals, whereas the MMSE can be used to detect MCI in elderly individuals aged 75 and older. Previously published studies have identified a clear ceiling effect for MMSE in the younger group, which may result in the low sensitivity of MMSE (18). However, as the age increases, age-related cognitive declines occur in older people, which may reflect a normal aging process (39). When the cognitive function of the elderly decreased significantly, both the MoCA and MMSE had lower sensitivities for detecting MCI, whereas the MMSE showed a higher specificity and could detect more severe cognitive failures (40).

According to recent studies, both the MoCA and MMSE scores were significantly influenced by the education levels of the study participants and were positively correlated with education levels (41, 42). These results are consistent with our study. The current cutoff score of the MoCA is not suitable for elderly individuals in China. For the samples with ≤ 12 years of education, it has been controversial whether adding 1 point to the score can adjust for the significant effect of education level on the MOCA scores of elderly Chinese people. Some studies have highlighted the need to establish different cutoff points for the MoCA for the samples with lower educational levels, similar to the MMSE (35, 38, 43). The MMSE and MoCA both significantly distinguished between normal cognition and dementia, which had high predictions for all three subgroups. The MoCA was found to be slightly more sensitive in screening for MCI than the MMSE in a subsample with higher education levels. For the lower education group, the abilities of the MOCA and MMSE to screen for MCI were low. Our results suggest that the MMSE has a limited ability to help identify MCI against CN individuals. Low education levels may affect the understanding of some MOCA tests. Individuals with lower education levels did not understand how to perform the “Alternating Trail Making” test (44). It has been reported in a study that, for Chinese people, 58.2% of subjects were unable to name the “rhinoceros” and “camel” in the “Naming tests,” and the words “velvet” and “church” were not easy to memorize in the “word memory” test. These words fall outside of the Chinese cultural background or the general understanding of Chinese people; therefore, there are difficulties for subjects with higher education, let alone lower education (43).

Both the MMSE and MoCA appeared to have similar performance levels in distinguishing between normal cognition and dementia. However, it was observed that for MCI in the more educated and younger participants, the MoCA had a substantially greater AUC and better specificity and sensitivity than the MMSE. This is consistent with the recognition that the MOCA has advantages in detecting subtle cognitive impairments when compared with the MMSE (45) and may indicate that the MoCA is more suitable as a screening tool for MCI in younger and higher educated Chinese elderly individuals. However, for the lower educated and older participants, the ability of the MMSE to detect MCI was significantly better than that of the MoCA, which means that the MMSE was more suitable for screening MCI in individuals with lower education levels and the older Chinese elderly.

Previous studies have suggested that the best detection of MCI can be achieved with a cutoff point of 24.5 for the MoCA, and a more important cutoff was 27.5 for the MMSE, which is among people with over 6 years of education and is not suitable for elderly Chinese population (46). The World Alzheimer's Report (2015 and 2018) estimated that 58% of people with dementia live in low and middle income countries. The Cut-off values of the cognitive scales currently used are not applicable for all the populations, as several subtests of the scales incorporate tasks may be influenced by education or literacy. This limitation of cognitive scales to discriminate MCI and dementia in elderly people has been previously reported both in developing countries and rural areas of developed countries (47, 48). Thus, we recalculated the cutoff value according to education level and age in this study, which has important clinical value for low-income and rural elderly individuals.

In our study, the optimal cutoff scores of the MoCA for the groups aged ≤ 75 years old and education ≤ 6 years, aged > 75 years old and education ≤ 6 years, aged ≤ 75 years old and education > 6 years, aged > 75 years old and education > 6 years in screening for MCI were identified as 19.5,15.5, 24.5, 24.5, respectively, and the optimal cutoff scores for dementia were 18.5, 10.5, 18.5, and 20.5, respectively. For the MMSE in the above four groups, the cutoff scores to detect MCI were 26.5, 22.5, 28.5, and 26.5, respectively, and the optimal cutoff scores for dementia were 23.5, 19.5, 23.5, and 23.5, respectively.

Thank you for your suggestion. In a meta-analysis exploring the use of the MoCA as a screening tool for MCI, studies including data from the original MoCA study revealed an optimal cutoff score of 23 (15). Analysis in a Chinese sample reported that differentiating between normal and impaired cognition for the total sample was 26.5 on the MMSE and 22.5 on the MoCA for MCI and 23.5 on the MMSE and 19.5 on the MoCA for AD (11), respectively. However, in work exploring the impact of education and age on cutoff for MCI or dementia, there are trends that the cutoff is lowered as age increases and education level decreases. In our research, we also found that among the younger and more highly educated Chinese elderly population, these are consistent with the cutoff scores of the MMSE and MoCA of previous studies, but they are not suitable for elderly populations that are older or have lower levels of education. The influence of education on the cutoff for MCI and dementia was more obvious, and the participants with lower levels of education had lower scores. In addition, the decreased cutoff score for Chinese elderly individuals with low education can improve the sensitivities and specificities of the MoCA and MMSE for detecting MCI and dementia. Our research examining the MoCA and MMSE stratified by age and education confirmed an optimal general cutoff score for cognitive screening. The cutoff score for MCI and dementia of the population aged > 75 years and education ≤ 6 years compensates for the shortcomings of previous studies.

Using biomarkers for diagnosing neurodegenerative illnesses in the elderly is expensive and invasive, and it is impractical to screen for cognitive impairment. MCI and dementia will be diagnosed based on detectable cognitive impairments, and simple cognitive screening, such as MOCA and MMSE, remains important. It is important that the cutoff criteria at different educational levels and ages are established to aid in the detection and diagnosis of neurodegenerative illness. Our results presented here provide the optimal cutoff scores for MCI and dementia for lower education elderly using the MoCA and MMSE, which are easily administered. Meanwhile, we have presented these results across different education levels and ages to guide screening decisions. Future work needs to be carried out in larger samples to examine the interaction between age and education and to establish the screening cutoff points for elderly Chinese individuals.

## Data Availability Statement

The raw data supporting the conclusions of this article will be made available by the authors, without undue reservation.

## Ethics Statement

The studies involving human participants were reviewed and approved by the Institution's Ethical Committee of Shanghai Mental Health Center, Shanghai Jiao Tong University School of Medicine. The patients/participants provided their written informed consent to participate in this study. Written informed consent was obtained from the individual(s) for the publication of any potentially identifiable images or data included in this article.

## Author Contributions

SwZ and XLi designed the study. JhW and FY offered significant comments on the manuscript. SfX, SxQ, XLin, FY, and LS are responsible for data collection and clinical diagnosis. SwZ and QQ interpreted and analyzed the data. SwZ wrote the first draft of the manuscript. All authors reviewed the manuscript.

## Funding

This work was supported by the National Key R&D Program of China (2017YFC1310500), the Shanghai Three-year Action Plan for Strengthening public Health System construction (2020-2022) (GWV-9.2), the Project of Shanghai Municipal Health Commission (2019SY045), and the Youth Scientific Research Project of Shanghai Municipal Commission of Health and Family Planning (20184Y0298).

## Conflict of Interest

The authors declare that the research was conducted in the absence of any commercial or financial relationships that could be construed as a potential conflict of interest.

## Publisher's Note

All claims expressed in this article are solely those of the authors and do not necessarily represent those of their affiliated organizations, or those of the publisher, the editors and the reviewers. Any product that may be evaluated in this article, or claim that may be made by its manufacturer, is not guaranteed or endorsed by the publisher.
